# The Bifunctional Enzyme SpoT Is Involved in the Clarithromycin Tolerance of Helicobacter pylori by Upregulating the Transporters HP0939, HP1017, HP0497, and HP0471

**DOI:** 10.1128/AAC.02011-16

**Published:** 2017-04-24

**Authors:** Xiwen Geng, Wen Li, Zhenghong Chen, Sizhe Gao, Wei Hong, Xiaoran Ge, Guihua Hou, Zhekai Hu, Yabin Zhou, Beini Zeng, Wenjuan Li, Jihui Jia, Yundong Sun

**Affiliations:** a Department of Microbiology, School of Medicine, Shandong University, Jinan, Shandong, People's Republic of China; b Department of Microbiology, Guiyang Medical University, Guiyang, Guizhou, People's Republic of China; c Laboratory of Experimental Teratology, Ministry of Education and Institute of Experimental Nuclear Medicine, School of Medicine, Shandong University, Jinan, Shandong, People's Republic of China

**Keywords:** Helicobacter pylori, SpoT, transporters, clarithromycin, antibiotic resistance

## Abstract

Clarithromycin (CLA) is a commonly recommended drug for Helicobacter pylori eradication. However, the prevalence of CLA-resistant H. pylori is increasing. Although point mutations in the 23S rRNA are key factors for CLA resistance, other factors, including efflux pumps and regulation genes, are also involved in the resistance of H. pylori to CLA. Guanosine 3′-diphosphate 5′-triphosphate and guanosine 3′,5′-bispyrophosphate [(p)ppGpp)], which are synthesized by the bifunctional enzyme SpoT in H. pylori, play an important role for some bacteria to adapt to antibiotic pressure. Nevertheless, no related research involving H. pylori has been reported. In addition, transporters have been found to be related to bacterial drug resistance. Therefore, this study investigated the function of SpoT in H. pylori resistance to CLA by examining the shifts in the expression of transporters and explored the role of transporters in the CLA resistance of H. pylori. A Δ*spoT* strain was constructed in this study, and it was shown that SpoT is involved in H. pylori tolerance of CLA by upregulating the transporters HP0939, HP1017, HP0497, and HP0471. This was assessed using a series of molecular and biochemical experiments and a cDNA microarray. Additionally, the knockout of genes *hp0939*, *hp0471*, and *hp0497* in the resistant strains caused a reduction or loss (the latter in the Δ*hp0497* strain) of resistance to CLA. Furthermore, the average expression levels of these four transporters in clinical CLA-resistant strains were considerably higher than those in clinical CLA-sensitive strains. Taken together, our results revealed a novel molecular mechanism of H. pylori adaption to CLA stress.

## INTRODUCTION

Helicobacter pylori is a helical or spiral-shaped Gram-negative bacterium that selectively colonizes the gastric mucosa. Most people worldwide are infected in early childhood and carry the organism throughout their lives (1). To date, H. pylori infection has been linked to human gastritis and gastric ulcers; moreover, long-term chronic infection by this pathogen increases the risk of gastric adenocarcinoma and mucosa-associated lymphoid tissue lymphoma (1, 2). Because H. pylori infection severely endangers human health, the treatment and eradication of H. pylori infection should be urgently addressed. Currently, the first-line treatment for H. pylori infection is a triple therapy that includes colloidal bismuth, proton pump inhibitors, and two antibiotics that are macrolides, nitroimidazoles, or β-lactam (3, 4). However, this traditional therapy does not work as effectively as before because of complex factors, such as the bacterium itself, host, treatment environment, and treatment methods (5). Currently, the antibiotic resistance of H. pylori is the principal reason for eradication failure (6). Nevertheless, the mechanism of resistance acquirement is not fully understood.

Clarithromycin (CLA) is the new generation of macrolide antibiotics. This drug is stable in acidic environments and well absorbed in the stomach (7). Consequently, CLA-based triple therapy has been recommended in children and adults by the latest Maastricht Consensus (8). One mechanism of H. pylori eradication is through CLA interference with protein elongation by binding reversibly to the peptidyl transferase loop of domain V in the 23S rRNA molecule, which could be induced to block bacterial protein synthesis (9, 10).

Recently, the drug resistance rate of H. pylori against CLA has been increasing worldwide because of the excessive and repeated use of CLA (4). The current research for CLA tolerance focuses on point mutations in the peptidyl transferase domain of the 23S rRNA ribosomal subunit (10). Recently, Hirata et al. reported that efflux pumps play a vital role in CLA resistance (11), and Smiley et al. completed a proteomic analysis and found that alterations in the outer membrane protein profile might be a novel mechanism conferring CLA resistance in H. pylori (12). Therefore, some other factors must be involved in CLA resistance in H. pylori.

SpoT regulates the stringent response in Escherichia coli (13). The stringent response is a bacterial adaptation that affects global gene expression during nutrient limitation and under other stress conditions (14). In E. coli, the stringent response is regulated by the small molecules guanosine 3′-diphosphate 5′-triphosphate and guanosine 3′,5′-bispyrophosphate [(p)ppGpp], which are synthesized by the enzymes RelA and SpoT. RelA and SpoT synthesize a considerable amount of (p)ppGpp using the substrates GDP and GTP and use ATP as a phosphorus source under nutrient limitation and other stressful conditions (13). These small-molecular effectors can combine with the RNA polymerase promoter (13–15) and subsequently change the promoter specificity and the efficiency of transcription (16).

Different from E. coli, the H. pylori genome contains only SpoT and lacks RelA (17, 18). SpoT is a bifunctional enzyme that has both synthetase and hydrolase activities, which are principal regulators for various stress responses in this bacterium (19–21). Recent studies have shown that (p)ppGpp mediates antibiotic tolerance in bacteria (22–24), which implies that antibiotic stress also activates the stringent response. For many gammaproteobacteria, microarray analyses of strains lacking ppGpp-synthesizing enzymes have revealed the deregulation of several hundred genes (25, 26). Thus, (p)ppGpp may be the universal signaling molecule regulator causing antibiotic resistance in H. pylori.

Multidrug efflux transporters are formed from the combination of a series of proteins, which can pump noxious compounds out of cells and protect cells from environmental stresses (27, 28). On the basis of sequence similarity, multidrug efflux transporters are classified into five families: the resistance-nodulation-cell division (RND) family, the major facilitator superfamily, the small multidrug resistance family, the multidrug toxic compound extrusion family, and the ATP-binding cassette (ABC) family (27, 28). Some identified multidrug efflux transporters are the AcrAB-TolC system in E. coli (29) and the MexAB-OprM system in Pseudomonas aeruginosa (30). H. pylori has four RND efflux transporters, and their participation in multidrug resistance has been confirmed (31–33). Nonetheless, many transporters are present in the H. pylori genome, and whether they participate in antibiotic tolerance should be determined.

Considering the important function of (p)ppGpp in the regulation of bacterial antibiotic resistance, the gradually increasing resistance to CLA in H. pylori, and the importance of transporters as factors in bacterial drug resistance, this study aimed to investigate whether (p)ppGpp is involved in the regulation of CLA resistance in H. pylori and to identify new transporters regulated by (p)ppGpp that participate in CLA resistance in H. pylori.

## RESULTS

### SpoT is involved in H. pylori resistance to CLA.

**FIG 1:**
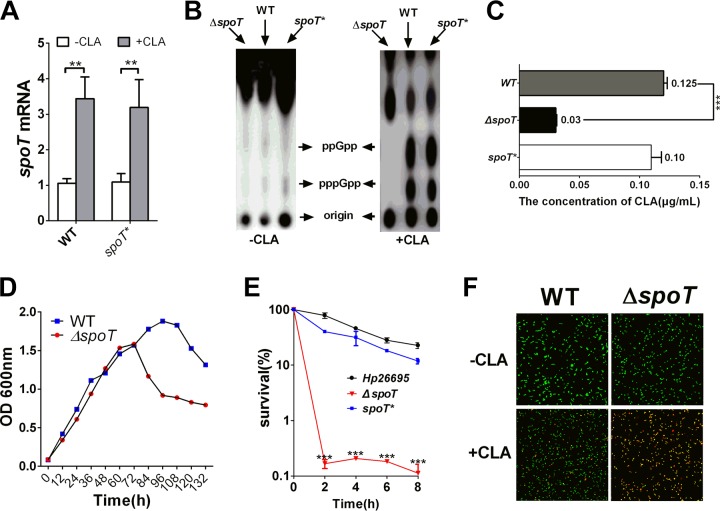
SpoT is involved in the resistance of H. pylori to clarithromycin (CLA). (A) CLA induces the high expression of the mRNA for SpoT in wild-type (WT) and SpoT-complemented (spoT*) strains. (B) H. pylori SpoT mutant strain (Δ*spoT*) is deficient in guanosine 3′-diphosphate 5′-triphosphate and guanosine 3′,5′-bispyrophosphate [(p)ppGpp] production when exposed to CLA (2× MIC) for 20 min. (C) MICs of WT, Δ*spoT*, and spoT* strains. The H. pylori Δ*spoT* mutant strain is sensitive to CLA. Complementation restores the MIC values. (D) Growth curves of the WT and Δ*spoT* strains. (E) Time-kill curves of WT, Δ*spoT*, and spoT* strains in the presence of 2× MIC of CLA. (F) Exposure to CLA (2× MIC, 2 h) induces coccoid transformation and the death of H. pylori cells. Cells stained with membrane-permeable Syto 9 (green) and membrane-impermeable PI (red) were visualized by confocal microscopy. The data are representative of three independent experiments and are shown as the means ± SEM from three independent experiments. Significance by *t* test: **, *P* < 0.01; ***, *P* < 0.001.

The bifunctional enzyme SpoT and (p)ppGpp are the key regulators in the stringent response of bacteria (16, 25). Moreover, the presence of CLA is unfavorable for H. pylori survival and growth. SpoT is involved in some bacterial antibiotic resistance. To determine whether SpoT participates in the regulation of the adaptation of H. pylori to CLA stress, the mRNA expression level of *spoT* was detected by quantitative real-time PCR (qRT-PCR). The expression of *spoT* in H. pylori strain 26695 (wild type [WT]) treated with CLA (1× MIC) was considerably higher (*P* < 0.01) than that in the control strain (Fig. 1A). SpoT is required for the production of (p)ppGpp. We subjected the H. pylori 26695 (WT), Δ*spoT* mutant, and *spoT*-complemented (spoT*; see Materials and Methods) strains to CLA stress, and they were incubated in minimal medium with ^32^P for 1 h. As expected, the WT and spoT* strains accumulated significant amounts of (p)ppGpp upon exposure to this stress. In contrast, (p)ppGpp was absent in the Δ*spoT* strain (Fig. 1B). We further examined the MIC of the WT, Δ*spoT*, and spoT* strains in response to CLA. The results showed that the MIC of the Δ*spoT* strain was only one-half of that of the WT strain (Fig. 1C). The viability of the Δ*spoT* strain was weaker than that of the WT strain after the stationary phase. In contrast, there was no significant difference in the exponential phase between these two groups (Fig. 1D). In time-kill experiments, we used cells in the exponential phase, and the results showed that the number of living cells of the Δ*spoT* strain decreased faster than the number of living cells of H. pylori 26695 and spoT* strains after treatment with 5× MIC of CLA for 2 h. After treatment for 8 h, the living cells of the Δ*spoT* strain decreased to 1% or less, but those of H. pylori 26695 and spoT* strains still made up more than 10%, which indicated that the Δ*spoT* strain is susceptible to CLA (Fig. 1E). Furthermore, we examined the cell membrane integrity of H. pylori cells and the morphological features of the WT and Δ*spoT* strains under CLA stress condition by applying membrane-permeable (Syto 9) and membrane-impermeable (propidium iodide [PI]) fluorescent dyes to stain living and dead cells, respectively. A larger number of cells of the Δ*spoT* strain than of wild-type cells died and lost cytoplasmic membrane integrity. Additionally, the Δ*spoT* strain transformed from its normal helical bacillary morphological features to coccoid features (Fig. 1F).

### The inactivation of SpoT decreases efflux activity.

**FIG 2:**
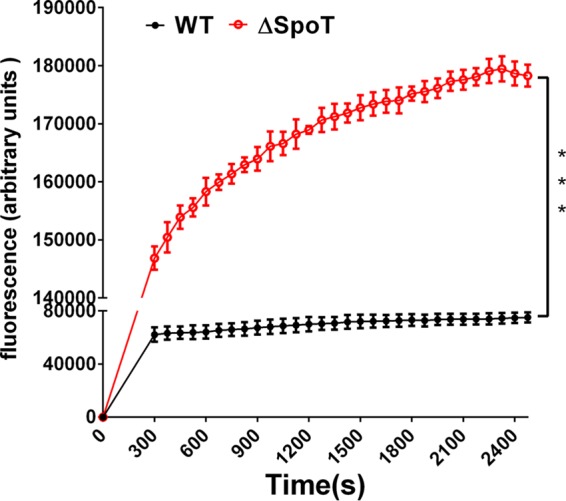
The accumulation of H33342 (2.5 μM) in H. pylori WT and Δ*spoT* strains. The fluorescence intensity was recorded at the excitation and emission wavelengths of 350 and 460 nm, respectively, over a 30-min incubation period. The data presented are the means ± SEM from three separate experiments. Student's *t* test was performed to compare the accumulation of Hoechst 33342 between the WT and Δ*spoT* mutant strains. ***, *P* < 0.001.

The results reported above indicated that SpoT contributes to the CLA stress response in H. pylori. Thus, we investigated its regulatory mechanism. Because previous studies have shown that SpoT regulates bacterial drug resistance via many aspects and efflux pumps are an important factor in multidrug-resistant bacteria, we assessed whether SpoT was involved in bacterial drug resistance by regulating the expression of efflux pumps in this study. The efflux activities of the Δ*spoT* and WT strains were assessed by determining the accumulation of the fluorescent dye Hoechst 33342 (H33342), and the results showed that SpoT inactivation caused an obvious increase in the accumulation of Hoechst 33342 (Fig. 2). These results indicated that some efflux pumps can be regulated by SpoT in H. pylori.

### cDNA microarray analysis was performed to detect the efflux pumps involved in H. pylori CLA resistance.

**FIG 3:**
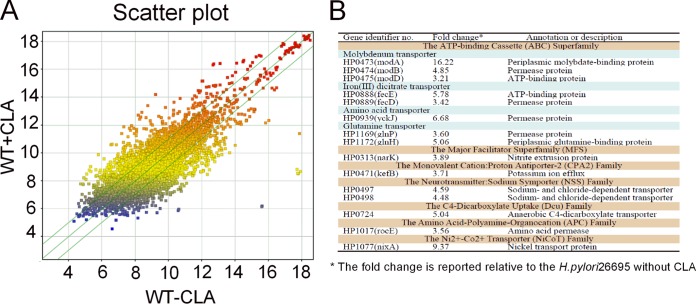
Microarray assay of the gene expression in H. pylori treated with CLA (0.125 μg/ml) for 2 h. (A) Scatter plot of the differentially expressed genes. The values on the *x* and *y* axes are the normalized signal values of the samples (log_2_ scaled). The green lines are the fold change lines. The default fold change value given is 1.5. The genes above the top green line and below the bottom green line indicated >1.5-fold change between the two compared samples. (B) Genes with >3-fold changes in H. pylori with CLA treatment in the microarray analysis. The microarray results represent a single experiment.

In general, because antibiotics are immediately drained, the genes encoding efflux pumps (proteinaceous transporters localized in the cytoplasmic membrane of all cells) in bacteria will be highly expressed under antibiotic pressure (34). To determine which efflux pump genes are involved in the resistance of H. pylori to CLA, we analyzed the differential expression of transporters between H. pylori strain 26695 cells treated with 2× MIC of CLA for 20 min and untreated cells (control) (Fig. 3A). The transporter genes that showed >3-fold-higher expression than the control were selected as potential target genes (Fig. 3B).

### qRT-PCR analysis was performed to identify the transporters that participate in H. pylori CLA tolerance.

**FIG 4:**
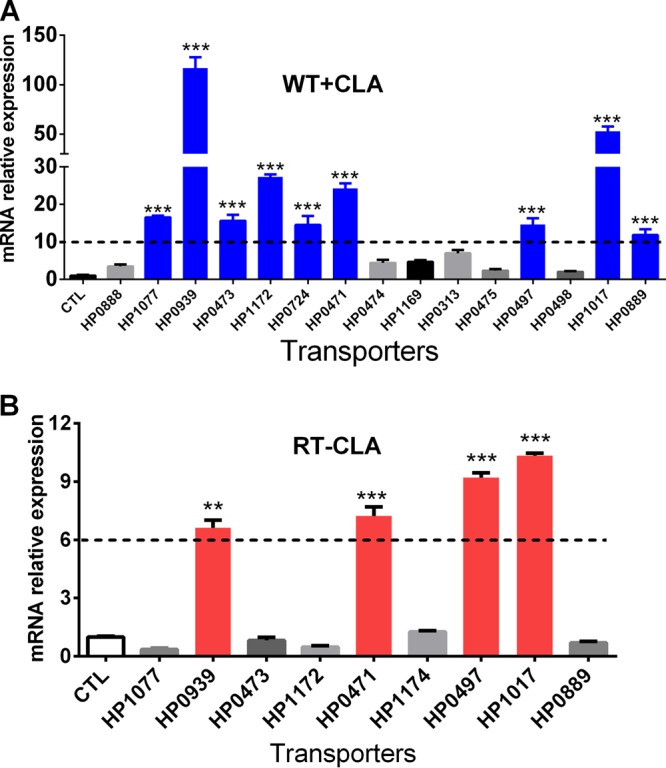
qRT-PCR analysis of the mRNA levels of transporters in H. pylori CLA-sensitive and -resistant strains. (A) qRT-PCR was used to confirm the expression of the transporters using cDNA microarray analysis (Fig. 3B) in a CLA-sensitive strain exposed to CLA for 2 h. The results were compared to those obtained in the absence of CLA treatment (CTL). Transporter expressions that increased >10-fold are in blue. (B) The transporters marked in blue in panel A were further analyzed by qRT-PCR in a CLA-resistant strain (ST, selected artificially) and compared to those of the CLA-sensitive strain without CLA treatment. Transporter expressions that increased more than 6-fold are in red (encoded by *hp101*7, *hp0939*, *hp0497*, and *hp0471*). The signals were normalized to the 16S rRNA levels. Data are means ± SEM from three independent experiments. Significance by *t* test: **, *P* < 0.01; ***, *P* < 0.001.

On the basis of microarray analysis, the selected transporters were further confirmed by qRT-PCR in the H. pylori 26695 WT strain exposed to 2× MIC of CLA for 2 h. We selected the transporters that were expressed >10-fold relative to those of the control (Fig. 4A). They were identified in a CLA-resistant strain (ST, selected artificially; MIC = 8 μg/ml). Sequence analysis of the 23S rRNA gene of the ST revealed mutations (data not shown), as shown by qRT-PCR. Only four genes (*hp0939*, *hp1017*, *hp0471*, and *hp0497*) were expressed significantly higher than in the control group (Fig. 4B). According to the analysis above, these four transporters are important for H. pylori adaptation to the CLA stress.

### The transporters HP0939, HP1017, HP0497, and HP0471 are involved in H. pylori resistance to CLA.

**FIG 5:**
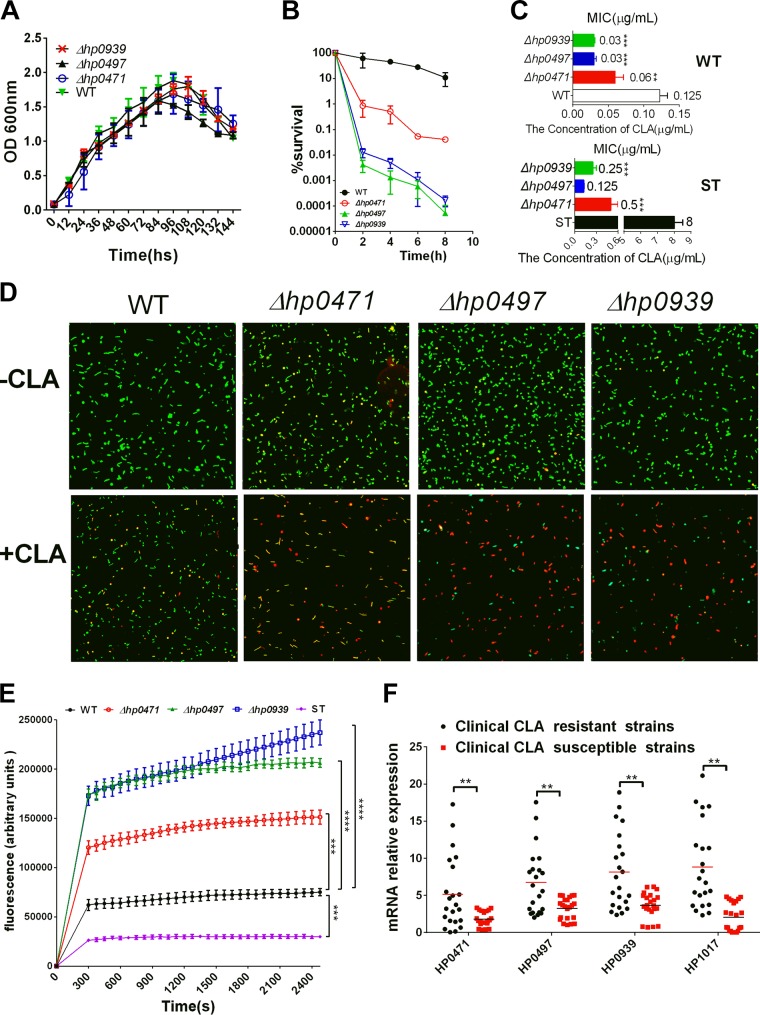
HP0939, HP0497, and HP0471 are involved in the resistance of H. pylori to CLA. (A) Growth curves of the WT strain and the Δ*hp0939*, Δ*hp0497*, and Δ*hp0471* mutants. (B) Time-kill assays in WT, Δ*hp0939*, Δ*hp0497*, and Δ*hp0471* strains with 2× MIC of CLA. (C) MICs of WT, ST, and their Δ*hp0939*, Δ*hp0497*, and Δ*hp0471* mutant strains. Compared with nonmutant strains, H. pylori transporter mutant strains are more sensitive to CLA. In ST mutant strains, the MICs of Δ*hp0939*, Δ*hp0497*, and Δ*hp0471* strains decreased considerably compared with those of the ST group. (D) Exposure to CLA (2× MIC, 2 h) induces coccoid transformation and the death of H. pylori cells. Cells stained with membrane-permeable Syto 9 (green) and membrane-impermeable PI (red) were visualized by confocal microscopy. (E) Accumulation of H33342 (2.5 mM) in the WT, ST, Δ*hp0939*, Δ*hp0497*, and Δ*hp0471* strains. The fluorescence intensity was recorded at the excitation and emission wavelengths of 350 and 460 nm, respectively, over a 30-min incubation period. The data presented are the means ± SEM from three separate experiments. Student's *t* test was performed to compare the accumulation of Hoechst 33342 in each strain with that in the WT. (F) qRT-PCR analysis of the mRNA levels of the H. pylori transporters (*hp1017*, *hp0939*, *hp0497*, and *hp0471*) between clinical CLA-resistant and -sensitive strains. Data are the means ± SEM from three independent experiments. Significance by *t* test: **, *P* < 0.01; ***, *P* < 0.001; ****, *P* < 0.0001.

The qRT-PCR data suggested that the transporters HP0939, HP1017, HP0497, and HP0471 may be involved in the resistance of H. pylori to CLA. Therefore, we constructed mutant strains for further study and examined the functions of these four transporters in drug resistance. However, we could not acquire a mutant strain for HP1017, which indicates that this transporter is essential for H. pylori survival. We successfully constructed *hp0939*, *hp0497*, and *hp0471* mutant strains in the WT and CLA-resistant strains (ST, artificially selected.). To test whether these transporters were required for H. pylori growth, we assayed the viability of the WT and mutant strains of these three genes (Δ*hp0939*, Δ*hp0497*, and Δ*hp0471* strains) in liquid cultures over 144 h under standard laboratory growth conditions. All strains exhibited similar population dynamics (Fig. 5A). In addition, the time-kill assays showed that the living cells of these three mutant strains exhibited a considerably sharper decrease than H. pylori (WT) after treatment with 5× MIC of CLA for 2 h. After 8 h of treatment, the living cells of these three mutant strains dropped to 0.1% or less, but those of the WT strain were still more than 10%. This observation indicated that these three mutant strains, and specifically Δ*hp0939* and Δ*hp0497* strains, were more susceptible to CLA than the WT strain (Fig. 5B). We further examined the MICs of these three mutant strains; the results showed that the MIC of the Δ*hp0471* mutant strain was only one-half that of the WT strain and the MICs of the Δ*hp0939* and Δ*hp0497* mutant strains were hardly one-quarter of that of the WT strain (Fig. 5C). Additionally, the MICs of ST and its Δ*hp0939*, Δ*hp0497*, and Δ*hp0471* mutant strains were also significantly decreased compared with those of the ST group; particularly, the MIC of the Δ*hp0497* strain was close to that of the WT strain (Fig. 5C). Moreover, we also examined the cell membrane integrity of H. pylori and the morphological features of the WT and these three mutant strains under CLA stress by applying the membrane-permeable (Syto 9) and membrane-impermeable (PI) fluorescent dyes to stain living and dead cells, respectively; a larger number of mutant cells than WT cells died and lost their cytoplasmic membrane integrity (Fig. 5D). As transporters, *hp0939*, *hp0497*, and *hp0471* were highly expressed after treatment with CLA, so we hypothesized that the transporters may discharge antibiotics. Subsequently, we assessed the efflux activities of these three mutant strains, the ST strain, and the WT strain by detecting the accumulation of the fluorescent dye Hoechst 33342. The result displayed that the inactivation of these three genes caused a distinct increase in the accumulation of Hoechst 33342. Notably, the fluorescence values of the Δ*hp0939* and Δ*hp0497* mutant strains were >4-fold greater than that of the WT (Fig. 5E). The relative mRNA expression levels of *hp0939*, *hp1017*, *hp0497*, and *hp0471* were assessed by qRT-PCR in the clinical CLA-resistant strains and the clinical CLA-sensitive strains. The average expression levels of these four genes in the clinical CLA-resistant strains were significantly higher than those in the clinical CLA-sensitive ones (Fig. 5F).

### The bifunctional SpoT significantly mediates the transcriptional levels of transporters HP0939, HP1017, HP0497, and HP0471.

**FIG 6:**
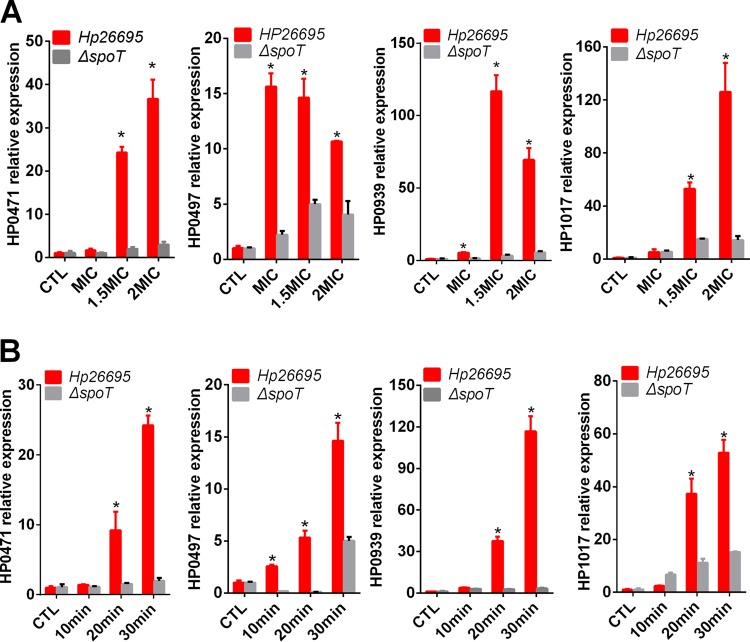
qRT-PCR analysis of the mRNA levels of transporters (*hp1017*, *hp0939*, *hp0497*, and *hp0471*) in WT and Δ*spoT* mutant strains exposed to different CLA concentrations for 30 min (A) and to 2× MIC CLA for different time periods (B). The results were compared to WT without CLA treatment (CTL). The signal was normalized to the 16S rRNA levels. *, *P* < 0.05 by *t* test.

The data reported above indicated that the transporters HP0939, HP1017, HP0497, and HP0471 were important for the adaption of H. pylori to CLA stress. Whether these transporters are regulated by the bifunctional SpoT was subsequently determined. First, we stimulated the WT and Δ*spoT* strains with 1.5× MIC of CLA along a time gradient (Fig. 6A). Second, we stimulated the WT and Δ*spoT* strains with different CLA concentrations (1.5× and 2× MIC) for 30 min (Fig. 6B). Compared with the control, the expression of all four transporters was highly induced by CLA in the WT strain in a concentration- and time-dependent manner. However, CLA could hardly induce their expression in the Δ*spoT* strain (Fig. 6A and B). These results suggested that these four transporters might be upregulated by SpoT in response to CLA stress.

### qRT-PCR was performed to analyze the expression of the efflux pumps (HefF and HefC) in the WT, Δ*hp0939*, Δ*hp0497*, Δ*hp0471*, and Δ*spoT* strains left untreated or treated with CLA.

**FIG 7:**
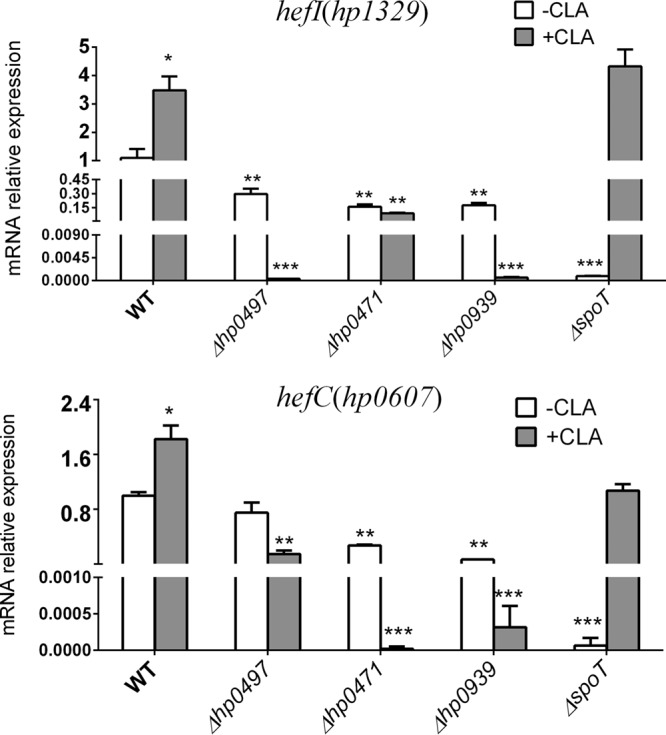
qRT-PCR analysis of the mRNA levels of efflux pumps (HefC and HefF) in WT, Δ*hp0939*, Δ*hp0497*, Δ*hp0471*, and Δ*spoT* mutant strains treated with or without CLA. The signal was normalized to the 16S rRNA levels. Significance by *t* test: *, *P* < 0.05; **, *P* < 0.01; ***, *P* < 0.001.

The accumulation of H33342 indicated that both SpoT (Fig. 2) and the transporter proteins (Fig. 5E) could affect the activity of the efflux pumps. However, we did not screen out efflux pumps by cDNA microarray analysis. Previous studies have shown that the efflux pumps of the RND family (HefF and HefC) are involved in H. pylori resistance to CLA (35). Therefore, we examined the expression of these two efflux pumps in the WT, Δ*hp0939*, Δ*hp0497*, Δ*hp0471*, and Δ*spoT* strains treated with 2× MIC of CLA or without CLA. The results showed that their expression levels in the mutant strains (Δ*hp0939*, Δ*hp0471*, and Δ*hp0497* mutants) were significantly lower than in the WT strain in both groups (Fig. 7). In the SpoT strain, the expression levels of these two efflux pumps were reduced in the absence of the antibiotic, but they were still induced by the addition of antibiotics (Fig. 7).

## DISCUSSION

In this study, SpoT, which participates in CLA resistance of H. pylori, was shown to be a global regulatory factor that might be involved in H. pylori antibiotic resistance in many ways. Our present study focused on its regulatory action on transporter proteins, and the results demonstrated that CLA stress could induce H. pylori to produce a large amount of (p)ppGpp, which contributes to the high expression of transporter protein genes. The regulation of transporter protein genes by SpoT might be one of the CLA-resistant mechanisms of H. pylori.

CLA is a common drug used in the first-line eradication therapy for H. pylori infection. Epidemiological studies have shown that the prevalence of CLA-resistant H. pylori has been increasing annually (9). Tolerance to CLA in H. pylori strains is often related to point mutations in two adjacent 23S rRNA nucleotides (A2143G or A2144G) that change the target region of CLA and decrease the affinity for 23S rRNA (10). The existing clinical data indicate that almost all CLA-resistant strains had 23S rRNA mutations (4, 11), including the strains used in the present study. However, our data demonstrate that transporter proteins (encoded by *hp0939*, *hp0471*, *hp0497*, and *hp1017*) are also important in H. pylori resistance to CLA, even in the resistant strains with 23S rRNA mutations. After knocking out the transporter genes (*hp0939*, *hp0471*, and *hp0497*), all strains exhibited a growth curve similar to that of the WT (Fig. 5A), but their sensitivity to CLA obviously increased. In our MIC determination study for the ST and its Δ*hp0497* mutant strain, the MIC of the Δ*hp0497* mutant was similar to that of the WT strain. This manifestation indicated that transporter proteins perform a crucial role in this drug resistance process in addition to point mutations.

Several efflux pump genes have been confirmed to participate in CLA tolerance (11, 35, 36). To avoid being killed by antibiotics, the bacteria achieve a lower intracellular drug concentration by lowering the rate of drug uptake or increasing the rate of drug export or both (34). In these experiments, the inactivation of these three genes (*hp0939*, *hp0471*, and *hp0497*) caused a distinctly increased accumulation of the dye Hoechst 33342. However, these four transporter proteins (HP0939, HP0471, HP0497, and HP1017) are not efflux pumps, and their predicted functions are the uptake of nutrients and the transport of ions. HP0939-0940 belongs to the YckJ amino acid ABC transporter family and may be related to amino acid uptake. The gene encoding HP1017 is similar to the *rocE* gene from Bacillus subtilis and encodes an arginase transporter (17). HP0497 is a sodium- and chloride-dependent transporter, and HP0471 (KefB) is a glutathione-regulated potassium efflux system protein (18). In short, the above-mentioned genes are involved mainly in the metabolism of H. pylori, and the physiological and metabolic states of bacteria can affect the efficacy of antibiotics (37, 38). Taking into account that these three transporters are not efflux pumps, the increased accumulation of the dye Hoechst 33342 in mutant strains may be attributed to either efflux or the uptake of the outer membrane affected by these mutants. A previous study reported that H. pylori had a less efficient self-promoted uptake pathway (31). In order to verify this, we examined the expression of the efflux pumps of *hefC* and *hefI* (RND family) with and without CLA treatment. The results showed that their expressions in the mutant strains (Δ*hp0939*, Δ*hp0471*, and Δ*hp0497* strains) were significantly lower than those in the WT strain (Fig. 7). It has been shown that these two efflux pumps are involved in H. pylori resistance to CLA (11, 35), which might be one of the most important reasons why these three mutants (*hp0939*, *hp0471*, and *hp0497* mutants) are more sensitive to CLA.

It can be seen in Fig. 5 that the mutation of any of the three transporters causes H. pylori to be sensitive to CLA. This finding suggested that the three gene products interacted in some way. To test this hypothesis, we analyzed the interactions among these transporters by qRT-PCR and found that after mutating one of these transporters, the expressions of the other transporters were suppressed compared with those in the WT strain (data not shown). This suggested that these four transporters might be regulated by similar mechanisms. In this study, we found that they were both regulated by SpoT (Fig. 6). Because SpoT is primarily involved in the stringent response of bacteria (23, 26), these four transporters might be important for the bacterial adaptation to environment stresses as well. Studies have shown that HP0497 is involved in the adaption of H. pylori to salt (39) and acid (40) stresses. According to the published H. pylori genome sequence of strain 26695, the HP1017 gene is an important part of the *htrA* locus (18). HtrA proteases and chaperones exhibit important roles in the stress responses (41). Moreover, there is evidence that HP0471 is involved in the H. pylori tolerance to CLA (42). Considering that the deletion of *spoT* did not increase the susceptibility of the resistant ST strain to CLA (data not shown) and these four transporters were highly expressed in CLA-resistant clinical isolates, genes encoding these four transporters might be potential candidate genes for H. pylori resistance to CLA.

In a comparison of the growth curves of the WT strain and SpoT resistant strain, the Δ*spoT* strain showed growth defects and was more likely to enter the decline phase (Fig. 1D), while no significant differences were found between the growth curves of the transporter mutant strains (Δ*hp0939*, Δ*hp0471*, and Δ*hp0497* strains) and the WT strain (Fig. 5A). This suggested that the Δ*spoT* strain was more sensitive to CLA than were the transporter mutants. However, the transporter mutant strains were more sensitive to CLA. In the transporter mutants, whenever they were exposed to CLA, the expressions of both of the efflux pumps (HefC and HefI) were inhibited (Fig. 7). However, in the Δ*spoT* strain, although the expression levels of these two efflux pumps were reduced in the absence of the antibiotic, they were still induced by the addition of antibiotics, which suggested that these two efflux pumps not only were regulated by SpoT under antibiotic stress but might also be controlled by other genes. This might be the reason why the transporter mutants were more sensitive to CLA than the Δ*spoT* strain. Alternatively, SpoT may affect the productions of HefC and HefI by regulating the expressions of these transporter genes (*hp0939*, *hp0471*, and *hp0497*), but this requires further study.

Acting as a global transcriptional regulator, (p)ppGpp enhances the bacterial ability to adapt to environmental pressures by controlling replication, transcription, translation, and metabolism (43, 44). Antibiotics are the most powerful weapons in the fight against bacteria, but they induce the stringent response and then result in (p)ppGpp accumulation. (p)ppGpp influences the transcription of genes associated with important metabolic pathways, which consequently affects various bacterial physiological processes, including resistance to antibiotics. (p)ppGpp is implicated in the resistance of bacteria to vancomycin (22), penicillin (45), and amdinocillin (46). A series of experiments demonstrated that (p)ppGpp affected bacterial resistance to drugs through diverse mechanisms (47). During the stringent response, (p)ppGpp inhibits peptidoglycan metabolism and induces bacteria resistant to penicillin (48, 49). (p)ppGpp can also mediate the upregulation of the YojI efflux pump and then enhance microcin resistance in E. coli (50). (p)ppGpp was synthesized by SpoT in H. pylori (17, 18), and SpoT is involved in H. pylori adaptation to nutritional deficiencies, oxygen, and acid stress (19–21). Our work extended those studies to CLA, a completely different kind of stress environment, and showed that the role of ppGpp in CLA resistance included the stimulation of the high expression of *hp0939*, *hp0471*, *hp0497*, and *hp1017* transporters, which would reduce the intracellular levels of the antibiotic by exporting CLA. However, the alarmone effect of ppGpp dependency *in vivo* is still unknown.

Although ppGpp activates the expression of transporters, such stimulation is only partial and perhaps insufficient to explain the function of ppGpp in the resistance of H. pylori to CLA. Therefore, more mechanisms need to be found to interpret all of ppGpp's effects. Thus, it can be suggested that mechanisms unrelated to efflux could potentially contribute to CLA resistance. CLA belongs to a group of macrolides that bind to the peptidyl transferase loop of domain V of the 23S rRNA molecule. This binding interferes with protein elongation and effectively blocks bacterial protein synthesis (9, 10). (p)ppGpp indirectly regulates translation by reducing the transcription of rRNA and protein genes, subsequently restraining the production of the building blocks for ribosome assembly (46). Additionally, rRNA is the target of CLA; thus, the inhibition of rRNA synthesis by (p)ppGpp may reduce the target of CLA, which is probably the reasons why (p)ppGpp is involved in the resistance of H. pylori to CLA.

In conclusion, this study discovered a new mechanism of H. pylori resistance to CLA. Admittedly, the specific regulatory mechanisms of (p)ppGpp for transporters still need further studies. With the present evidence, our study provides support for the clinical treatment and epidemiological investigation of drug resistance in H. pylori.

## MATERIALS AND METHODS

### Bacterial strains, media, growth conditions, clinical isolation of H. pylori, determination of the MIC, and detection of mutations.

The H. pylori strains used in this study were reference strain 26695, which was kindly provided by Zhang Jianzhong (Chinese Disease Control and Prevention Center), and its isogenic *spoT* mutant, which was used in our previous report and comes from the collection of our laboratory (52). All E. coli strains used were DH5α and TOP10 (TransGen Biotech, Beijing, China). H. pylori strains were cultivated on Skirrow agar plates with 5% (vol/vol) sheep blood at 37°C and in a microaerobic environment (5% O_2_, 10% CO_2_, and 85% N_2_). The liquid culture medium for H. pylori consisted of brucella broth (BB) containing 10% fetal bovine serum (FBS), and the cells were incubated at 37°C in a shaker set at 120 rpm. The mutant strains were supplemented with kanamycin (Sigma-Aldrich, St. Louis, MO) to 25 μg/ml. E. coli strains were grown in Luria-Bertani medium at 37°C. Ampicillin and CLA were obtained from Sigma-Aldrich (St. Louis, MO).

Twenty-two CLA-resistant and 22 CLA-sensitive clinical isolates were obtained from patients, including those with gastritis, gastric ulcers, and duodenal ulcers and gastric cancer patients, at Qiannan People's Hospital (Guizhou Province). All patients provided informed consent before examination. The MICs of all the clinical and standard strains for CLA were determined by Etest as well as by the agar dilution method reported by Osato et al. (51). The bacteria (optical density at 600 nm [OD_600_], 0.8) were inoculated on an agar plate containing 2-fold dilutions of CLA (0.0156 to 1 μg/ml). All the plates were incubated at 37°C under microaerobic conditions, and the MIC values were determined. For resistant strains, PCR amplification of the 23S rRNA and sequence analysis were performed to examine the genetic basis of these resistant phenotypes. The above-described experiment details have been previously reported in a published article (53).

### Construction of *hp0939*, *hp1017*, *hp0497*, and *hp0471* mutant and *spoT*-complemented strains.

The plasmids pILL570 and pUC18K2 were kindly provided by Agnès Labigne (Unité de Pathogénie Bactérienne des Muqueuses, Institut Pasteur). The construction of *hp0939*, *hp1017*, *hp0497*, and *hp0471* mutant strains was identical to the construction of the Δ*spoT* strain, as described in the literature (52). Briefly, these four genes from the genome of H. pylori 26695 (wild-type and artificially selected resistance strains) were destroyed with the insertion of the nonpolar *aphA-3* gene encoding a kanamycin resistance cassette (54). All primers used in these studies are listed in Table 1.

**TABLE 1:** Primers used in this study^*a*^

Forward primers		Reverse primers	
Name	Sequence (5′–3′)	Name	Sequence (5′–3′)
hp1017XF	AACTGCAGAACCGTTTCTCAAGTCGTGG	hp1017XR	CGGAATTCGCGATGTATTCTAACGCCACC
hp1017SF	CGGGATCCAGCCCTTTTGTGAGCGTTTT	hp1017SR	CCATCGATTTGGGCATGCTCTGGATTTG
hp0939SF	CCATCGATGCCCCATGTTTGAAAAGCCT	hp0939SR	CGGGATCCACGCCAAGCTTGAGTAACAC
hp0939XF	CGGAATTCGGCGCGATTTTAATGAGAGCT	hp0939XR	AACTGCAGTGATGTGGAAGTGGCTAGGG
hp0471SF	CCATCGATATTGAGCGTAAGCCATCACC	hp0471SR	CGGGATCCACCTCTTCTTGGCCCTTTTT
hp0471XF	CGGAATTCTGGGAATGGCTGCAATATCT	hp0471XR	AACTGCAGCCATTCGCTCTTCTTCATCC
hp0497XF	AACTGCAGCGCCTTCAGAGTCGGTATCT	hp0497XR	CGGAATTCGCCCTATGGATTGTAGCCCT
hp0497SF	CGGGATCCTATGGGGCGAATGTCTCACA	hp0497SR	CCATCGATATGAGAGGGCCGCCTAAAAT
hp1172F	AGTCGCTTTAGGGGTGGTTT	hp1172R	AGGGTGTTGTTTCGTCCAAG
hp1169F	ATCGGTTTGAGCGCTTTAGA	hp1169R	AAATCACATTCGCCCCTATG
hp0473F	GAAAAGCCAGCATGGAAGTC	hp0473R	GGGTTGTTAGCCCCATTTTT
hp0888F	CGTTCAATTTCAGCGTGCTA	hp0888R	AAGCACCATTTGCCTTTGAC
hp0724F	CGCTCATTCAAGCGTGTTTA	hp0724R	GGATGAGCGCTTTAGAGGTG
hp1077F	TTTCTATGGGGCATTCAAGC	hp1077R	TTGTTGCTGGCTTAGGCTTT
hp0939F	CACGCCAAGCTTGAGTAACA	hp0939R	ATCAAAGCGGCTTCCAAATA
hp0475F	CGAAAAATCGGCTTTGTGTT	hp0475R	GGCTGCGATTAAAGCTCTTG
hp0498F	GCCGACAATTTGGTTTGTTT	hp0498R	TGCCCTAAAGCGTCCATAAC
hp0313F	CGCATGCTTTTTACCCATTT	hp0313R	AGAAAACACCACCCACGAAG
hp0724F	CACCTCTAAAGCGCTCATCC	hp0724R	AAACCCCAGGGATCAAAAAC
hp0471F	TGGGGATTTTGATTTTCCAA	hp0471R	GCGCTGCAAACAATCACTAA
hp0497F	TTGCTGGCATCACTTCTACG	hp0497R	TGCAAAATCCAACCAATCAA
hp1017F	ATGGGCTGTCCAAACAAAAG	hp1017R	TGCGAAACAGACACGCTAAC
hp0889F	TAATTCCGTCCTTTCGTTGG	hp0889R	AATTGATGCGCCACCTTAAC
hp0607F	GTTCGCCCTTCCAAACCTTT	hp0607R	CGCTCACGCCGTATTTGTTA
hp1329F	TGCCCCTTTAGCTTACACCA	hp1329R	TAAACCCAGGACGCTTGCTA

a Underlining indicates nucleotides that were added at the 5′ end to create a restriction site.

The *spoT* complement was constructed using the chloramphenicol resistance cassette from pMcagA, kindly provided by Wei Hong (Department of Microbiology, Guizhou Medical University, China). Full-length *spoT* was cloned into pMcagA, and the resulting plasmid was inserted into the middle of the *hp0547* (*cagA*) gene, which provided homologous recombination sites in H. pylori. The vector-transformed *spoT* mutant strain was constructed by electroporation to obtain the *spoT*-complemented strain (spoT*). The genotype of the complemented spoT* transformant was verified by PCR and the sequencing of the genomic loci.

### Detection of (p)ppGpp accumulation patterns.

The (p)ppGpp production was assayed as previously described (20, 22). Briefly, H. pylori strains were grown overnight in brucella broth containing 10% FBS to the early exponential phase (OD_600_, approximately 0.4), diluted back to an OD_600_ of 0.2, and incubated for an additional 2 h. When all strains reached an OD_600_ of approximately 0.3, samples (OD_600_, approximately 0.3) of each culture were removed and pelleted via centrifugation at 10,000 rpm for 5 min, after which samples were resuspended in 250 μl of liquid culture medium. ^32^P (Amersham) was added to 100 μCi ml^−1^, and the cultures were labeled for 2 h at 37°C. Subsequently, the experimental cells were treated with CLA (0.12 μg ml^−1^) for 1 h. Aliquots that were labeled for the duration of the experimental cultures were used as controls. After treatment, 50 μl of samples was added to an equal volume of 2 M formic acid. Afterward, at least four freeze-thaw cycles were conducted. The acid extracts were centrifuged briefly, and the supernatant fluids were spotted onto polyethyleneimine-cellulose plates (Sigma-Aldrich), dried, and developed in 1.5 M KH_2_PO_4_ (pH 3.4) for approximately 2.5 h. The results were obtained using phosphor screen scanning (Bio-Rad).

### Time-kill assay and growth curves.

Time-kill curve analyses were performed by culturing H. pylori in brucella broth medium. Plate-grown bacteria were cultured for 48 h under microaerobic conditions, inoculated into brucella broth with a preliminary OD_600_ of 0.08, and cultured for another 36 h with shaking at 120 rpm. CLA was added to the broth to a final concentration of 0.6 μg/ml. The CFU were counted at different time intervals (0, 2, 4, 6, and 8 h). Each experiment was repeated at least three times, and the raw data were analyzed using Excel.

To determine the involvement of knockout genes (*hp0939*, *hp0471*, *hp0497*, and *spoT*) in growth when strains were subjected to the same conditions, the growth kinetics of mutant strains were compared with those of the WT. The growth profiles were monitored in brucella broth with a preliminary OD_600_ of 0.08 and then cultured for another 144 h at 37°C with shaking. The records were taken every 12 h by determining the OD_600_ of the test strains. The values stated are the mode values from at least three biological replicates performed in at least three independent occasions.

### H33342 accumulation assay.

Accumulation assays were performed as described previously with slight modifications (55). Strains were inoculated in fresh medium and cultured under microaerobic conditions for 48 h at 37°C. The cells were harvested by centrifugation at 6,000 × g for 5 min at room temperature and subsequently suspended in phosphate-buffered saline (PBS). The final OD_600_ of the suspensions was adjusted to 0.1, and aliquots (180 μl) were transferred into a 96-well plate. The excitation and emission were measured at 355 and 460 nm, respectively, using FLUOstar Optima (Aylesbury, UK). Recordings were started 5 min after the addition of H33342 (25 μM, 20 μl). Readings were taken every 75 s for 30 cycles, and the raw data were analyzed using Excel. Each experiment was repeated at least three times.

### Microarray analysis.

The total RNA was extracted from H. pylori left untreated or treated with CLA (0.25 μg/ml) for 20 min using TRIzol reagent (Invitrogen, Carlsbad, CA). For microarray analysis, an Agilent array platform was employed by Shanghai Kangcheng Sheng Biological Engineering Co., Ltd. (China). The sample preparation and microarray hybridization were performed based on the manufacturer's standard protocols. Briefly, 1 μg of total RNA from each sample was amplified and transcribed into fluorescent cRNA using the manufacturer's Agilent's Quick Amp Labeling protocol (version 5.7; Agilent Technologies). The labeled cRNAs (Cy3) were hybridized onto the whole H. pylori 26695 Genome Oligo Array (8×15K; Agilent Technologies). After the slides were washed, the arrays were scanned by the Agilent Scanner G2505B. The Agilent Feature Extraction software (version 10.7.3.1) was used to analyze acquired array images. Quantile normalization and subsequent data processing were performed using the GeneSpring GX v11.5.1 software package (Agilent Technologies). After quantile normalization of the raw data, the genes with at least 2 of 2 samples having flags in “Present or marginal” (“All Targets Value”) were used for further data analysis. Differentially expressed genes between the two samples were identified through fold change filtering (fold change ≥ 1.5). The microarray results represent an independent single experiment.

### qRT-PCR.

The total RNA was extracted using TRIzol reagent (Invitrogen, Carlsbad, CA). The total RNA (1 μg) was reverse transcribed using the PrimeScript RT Reagent kit with gDNA Eraser (TaKaRa). The primers are shown in Table 1. The resulting cDNA was diluted, and 5 μl of this dilution was used in 20-μl qPCR mixtures containing 0.8 μl of the primer mixtures, 10 μl of SYBR Premix Ex TaqTM (TaKaRa, Otsu, Shiga, Japan), and 4.2 μl of double-distilled water. RT-PCR was done using the ABI Prism 7000 Sequence detection system (Applied Biosystems, Carlsbad, CA) for 1 cycle at 95°C for 30 s and 40 cycles at 95°C for 5 s and 60°C for 31 s. Dissociation curve analysis was performed to verify product homogeneity. The 16S rRNA amplicon was used as an internal control for data normalization. The changes in transcript level were determined by applying the relative quantitative method (ΔΔ*C*_*T*_). The threshold cycle (*C*_*T*_) values from all three biological replicates for each strain were compiled. The primers used are listed in Table 1.

### Confocal microscopy.

For confocal microscopy, we used the same method as the one described in our previous work (56). To determine bacterial shape and viability, bacterial cells were stained with membrane-permeable and membrane-impermeable fluorescent dyes from the Live/Dead BacLight Bacterial Viability kits (Molecular Probes, Invitrogen, USA) and were observed by confocal microscopy. The H. pylori cells were collected, washed, resuspended in BB liquid medium, and inoculated to the desired optical density at OD_600_ into BB liquid medium buffered with 10 mM sodium phosphate (pH 6.3). In addition, the cells were supplemented with 10% newborn calf serum or pure water (preliminary OD_600_, 0.05) and grown under microaerobic conditions. Aliquots were taken at different time points. They were stained with Syto 9 and propidium iodide (PI) for 15 min and washed twice with PBS. The cells were spread on glass slides, covered with mounting medium and coverslips, and visualized by confocal microscopy (Leica TCS SP5; Leica Microsystems GmbH, Wetzlar, Germany). Syto 9 is a green fluorescent membrane-permeable dye that labels all bacteria by staining nucleic acid, whereas PI is a red fluorescent membrane-impermeable dye that labels only bacteria with damaged membranes.

### Statistical analysis.

The data are presented as the means ± standard errors of the means (SEM). Statistical significance was determined using an unpaired Student's *t* test, and the *P* values were corrected by the Sidak-Bonferroni method for multiple comparisons. *P* values of <0.05 were considered statistically significant. The results were analyzed using the GraphPad Prism software (GraphPad Software Inc., La Jolla, CA, USA).

### Sequence accession number(s).

The microarray data obtained in this study were deposited in the Gene Expression Omnibus database (accession no. GSE84501).
